# Dying to know: does performance-based financing reduce mortality?

**DOI:** 10.1093/heapol/czab125

**Published:** 2021-10-16

**Authors:** I Bonfrer

**Affiliations:** Erasmus School of Health Policy & Management, Erasmus University Rotterdam, P.O. Box 1738, Rotterdam 3000 DR, The Netherlands

Arguably the most important unanswered question in the field of performance-based financing (PBF) in low-income countries (LICs) concerns its effects on health outcomes. A recently published study in this journal by Gage and Bauhoff (Gage and Bauhoff, 2021) sets out to answer this question by asking: What is the effect of PBF on neonatal mortality and low birth weight in Burundi, Lesotho, Senegal, Zambia and Zimbabwe? As an estimated 1 150 000 infants die each year during pregnancy and childbirth in LICs (The World Bank, 2021b), massive health gains could be realized if PBF proves to be effective in reducing mortality.

## Performance-based financing

PBF is the provision of financial incentives to health care providers with the aim of improving outputs or outcomes. These incentives are often introduced in LICs to improve maternal and child health (The World Bank, 2021a). The rationale is that health workers will increase their efforts to provide high-quality care if this means they can earn more money. These financial incentives are often supplemented with PBF programme components to actively involve local communities, increase the autonomy of health care facilities, and reduce inequities (Bonfrer *et al.*, 2014).

## Effects on mortality

The PBF approach in low- and middle-income countries has been supported with large investments from the World Bank through its Result Based Financing programme (Fritsche *et al.*, 2014) across more than 30 countries (The World Bank, 2021a). The evidence base on the effectiveness of this approach is growing, but two recent systematic literature reviews show that reliable estimates of PBF’s effects on mortality are not yet available (Diaconu *et al.*, 2021; James *et al.*, 2020).

## PBF programs across five countries

Gage and Bauhoff (Gage and Bauhoff, 2021) use publicly available Demographic and Health Survey and Multiple Indicator Cluster Survey data from Burundi, Lesotho, Senegal, Zambia and Zimbabwe to estimate a difference-in-differences model to determine the effects of PBF. The authors pool the data from these five countries for their main analyses and then estimate effects for each country individually and for a subset of poor or high-risk women.

## Null effect and required sample size

Gage and Bauhoff (Gage and Bauhoff, 2021) conclude that the five PBF programmes they evaluated had *no effect* on neonatal health outcomes—neither in the five countries combined, nor individually, nor among poor or high-risk women. I argue that Gage and Bauhoff do not provide convincing evidence of the claimed null effects. The reason is that, even in the pooled dataset, the sample sizes are too small to reliably estimate statistically significant effects on neonatal mortality.

Power analysis can be used to determine the sample size that is required to observe a statistically significant difference in an outcome between a treatment group and the control group when such a difference actually exists (Moffatt, 2021). After setting *α*, the probability of rejecting the null hypothesis when it is true (Type I error), and *β*, the probability of failing to reject the null hypothesis when it is false (Type II error), the power analysis can be applied (Moffatt, 2021). Gage and Bauhoff (Gage and Bauhoff, 2021) performed a power analysis, setting *α* and *β* to the conventional values of 0.05 and 0.8, respectively, and they report the minimum detectable effect (MDE) sizes (Appendix 5). For early neonatal death, the MDE is smaller than the effect they report (Table 4), suggesting that even their largest sample—the pooled data—is not sufficiently powered to identify a statistically significant null effect. In other words, given the sample sizes that Gage and Bauhoff report (13 164 for the intervention group and 18 484 for the control group), the resulting power of their estimation is 0.05, suggesting only a 5% chance that the test will reject the null hypothesis when an alternative hypothesis (PBF does affect neonatal mortality) is true.

To show that PBF would have a small but relevant effect on neonatal mortality—say, a 1 percentage point reduction—we would need a sample of more than 300 000 observations (assuming *α* = 0.05 and *β* = 0.8), half of these in the intervention group and half in the control group. Given that Demographic and Health Surveys range from about 5000 to 30 000 observations, it is unlikely that these can serve as a relevant source of data to estimate the effect of PBF on neonatal mortality, unless the expected effect size is considerably larger. Administrative data, if they are available and reliable, are likely to be better suited to estimate the mortality effects of future interventions, as they would allow a much larger number of observations.

## Heterogeneity

Even if we were to accept the null effect, building on the authors’ suggestion (p. 7) that with a larger sample size an effect would still not be detectable, the claim that PBF has no effect on neonatal mortality is disputable. The authors indicate that the PBF interventions included in their study ‘differed in their design and implementation across the five study countries’ and ‘varied widely in the number and type of indicators’ that were used to incentivize health care providers. Therefore, at least in theory, the five heterogeneous PBF programmes from locations more than 10 000 km apart could have very different effects on neonatal mortality. If the effects were to go in opposite directions, as the non-statistically significant positive and negative country estimates might suggest (Table 3 in Gage and Bauhoff), these would cancel each other out, potentially masking effects of PBF on mortality.

## Unclear effects on neonatal mortality

Although the conclusion that PBF had no effect on health outcomes may be correct, it is not warranted based on this study. The authors do briefly note that their study may not be adequately powered to detect changes in early neonatal death (p. 7) but then go on to conclude that they found no statistically significant impact of PBF on neonatal health outcomes. This does not seem internally consistent. The authors have also shared these findings with a broader audience of health care professionals and policy makers using, among others, Twitter, where the first author stated that ‘Across the five programs, we found no impact on the health outcomes or health system outputs’ and then attempted to explain what these ‘null effects’ mean (Gage, 2021). I agree that involving a broader audience is essential. However, while many are dying to know whether PBF can indeed provide improvements in health outcomes, the evidence to date does not provide credible insights into its effects on neonatal mortality.

## Data Availability

No new data were generated or analysed in support of this research.
